# Enterohemorrhagic *Escherichia coli* Effector Protein EspF Interacts With Host Protein ANXA6 and Triggers Myosin Light Chain Kinase (MLCK)-Dependent Tight Junction Dysregulation

**DOI:** 10.3389/fcell.2020.613061

**Published:** 2020-12-23

**Authors:** Ying Hua, Jiali Wu, Muqing Fu, Jinyue Liu, Xiaoxia Li, Bao Zhang, Wei Zhao, Chengsong Wan

**Affiliations:** ^1^Biosafety Level 3 Laboratory, Department of Microbiology, School of Public Health, Southern Medical University, Guangzhou, China; ^2^Key Laboratory of Tropical Disease Research of Guangdong Province, Guangzhou, China

**Keywords:** EHEC, O157: H7, EspF, protein–protein interaction, ANXA6, tight junction breakdown

## Abstract

Enterohemorrhagic *Escherichia coli* (EHEC) O157:H7 is an important foodborne pathogen that can cause bloody diarrhea and hemolytic uremic syndrome (HUS) in humans. EspF is one of the best-characterized effector proteins secreted from the type three secretion system to hijack host cell functions. However, the crucial pathogen-host interactions and the basis for the intestinal barrier disruption during infections remain elusive. Our previous study screened and verified the interaction between host protein ANXA6 and EspF protein. Here, by fluorescence resonance energy transfer (FRET) and co-immunoprecipitation (CO-IP), we verified that EspF interacts with ANXA6 through its C-terminal domain. Furthermore, we found that both the constitutive expression of EspF or ANXA6 and the co-expression of EspF-ANXA6 could decrease the levels of tight junction (TJ) proteins ZO-1 and occludin, and disrupt the distribution of ZO-1. Moreover, we showed that EspF-ANXA6 activated myosin light chain kinase (MLCK), induced the phosphorylation of myosin light chain (MLC) and PKCα, and down-regulated the expression level of Calmodulin protein. Collectively, this study revealed a novel interaction between the host protein (ANXA6) and EspF. The binding of EspF to ANXA6 may perturb TJs in an MLCK-MLC-dependent manner, and thus may be involved in EHEC pathogenic function.

## Introduction

Enterohemorrhagic *Escherichia coli* (EHEC) O157:H7 is an important foodborne pathogen; it has been linked to a broad spectrum of diseases ranging from bloody diarrhea to hemorrhagic colitis and even life-threatening hemolytic uremic syndrome (HUS) (Tarr et al., 2005). EHEC-associated enteritis and diarrhea are closely related to the subversion of intestinal mucosal epithelial cells barrier integrity and the disruption of epithelium permeability (Roxas et al., 2010). However, the underlying mechanisms are yet to be elucidated.

One of the characteristics of EHEC infection is the increased permeability of solutes through intestinal epithelium (Garmendia et al., 2005). The intestinal epithelial cells (IECs) are held together through an adhesive complex comprising tight junctions (TJs), adherens junctions, and desmosomes (Tsukita et al., 2001). TJs are complex structures located in the most apical region of the lateral membrane and modulate two primary functions in epithelia (van Itallie and Anderson, 2014). First, they form a physical barrier that restricts paracellular transportation of molecules such as ions, solutes, water, and immune cells (Pawlowska and Sobieszczanska, 2017). Furthermore, TJs act as a fence to limit the movement of lipids and intimal proteins between the apical and basolateral membranes, thereby creating a barrier function and cell polarity (Turner et al., 2014). The transmembrane barrier proteins, occludin, and claudin, as well as peripheral scaffolding protein, ZO-1, are the main functional proteins of TJs (Runkle and Mu, 2013). Claudin and occludin are responsible for the paracellular permeability barrier. ZO-1 has been implicated in linking the cytoskeleton and TJs. These proteins maintain TJ structure and function by interacting with the actin cytoskeleton; meanwhile, this interaction allows the cytoskeleton to manipulate TJ barrier integrity (van Itallie et al., 2009; Gunzel and Fromm, 2012). Myosin light chain kinase (MLCK), which induces contraction of the perijunctional apical actomyosin ring in response to the phosphorylation of the myosin light chain (MLC), gives rise to the opening of the paracellular pathways (Cunningham and Turner, 2012), thus playing a pivotal role in regulating TJ permeability. Therefore, it is not surprising that TJs are one of the main targets of bacterial proteins and key players in host-pathogen interaction.

Once enteric bacterial pathogens infect IECs, there is a redistribution of TJ proteins, which destroys the TJ structure and barrier function (Awad et al., 2017). The disruption of tight junction integrity, which contributes to diarrhea, is critical to the pathogenic mechanism of EHEC and enteropathogenic *E. coli* (EPEC) (Clements et al., 2012). Multiple signal transduction pathways regulate TJs. Among these, the MLCK, phospholipid kinase C (PKC), and mitogen-activated protein kinase (MAPK) pathways play vital roles in the regulation of TJ structure by modulating TJ protein assembly, decomposition, and phosphorylation (Gonzalez-Mariscal et al., 2008). MLC can serve as a substrate for the Ca^2+^ activated, phospholipid-dependent PKC, as well as for the Calmodulin-dependent MLCK (Dahan et al., 2003). PKC can activate MLCK, causing MLC phosphorylation, actin contraction, and cytoskeletal remodeling, which may destroy TJs (Turner et al., 1999). Studies have demonstrated that EPEC induces MLC phosphorylation, and the transepithelial electrical resistance (TER) decrease can be inhibited by the MLCK inhibitor ML-9 (Yuhan et al., 1997). However, the effect of EHEC infection on MLC phosphorylation has not been investigated. EPEC was reported to facilitate the collapse of TJ by declining TER, increasing the monolayer permeability, relocalizing ZO-1, dephosphorylating occludin, and redistributing claudin (Ugalde-Silva et al., 2016). Like EPEC, EHEC can also elicit a TER loss, which is independent of the Shiga toxin. Furthermore, a murine model of infection by EHEC also indicated a rapid disruption of the TJ fence function (Roxas et al., 2010). Although the mechanism underlying TJ disruption induced by EHEC/EPEC remains unclear, extensive studies have suggested that EspF may play an important role (Mcnamara et al., 2001; Tapia et al., 2017a; Singh et al., 2018; Xia et al., 2019).

**Figure 1:**
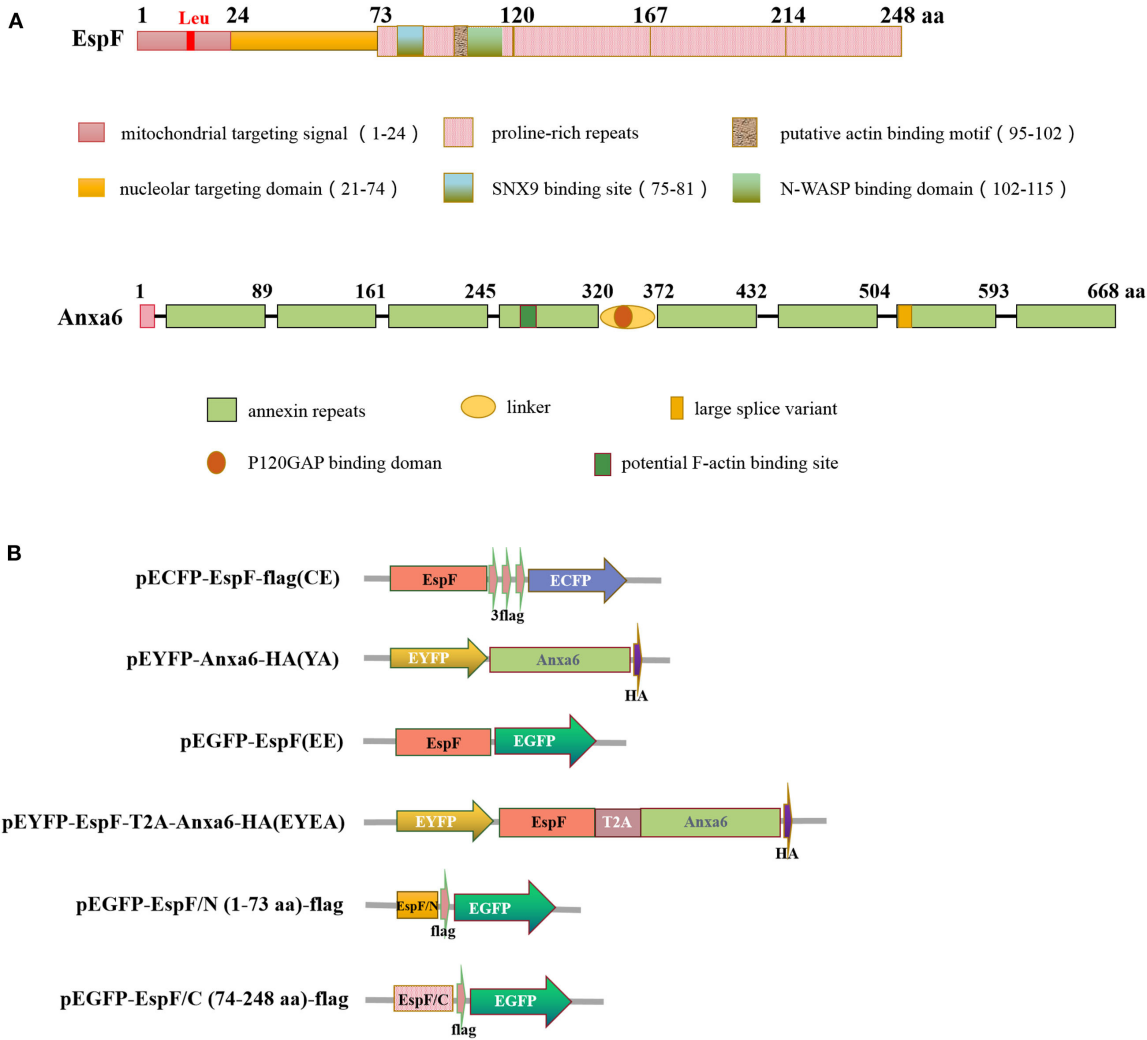
Diagram of EspF protein and ANXA6 protein structure **(A)** and plasmid constructs used in this study **(B)**. aa, Amino acid.

EspF is one of the most significant effector proteins of EHEC and EPEC, which is injected into host cells through a molecular syringe termed type III secretion system (T3SS) after the colonization of EHEC/EPEC to IEC (Holmes et al., 2010). The N-terminal region of the EspF protein is highly conserved and contains a secretory signal that can aid EspF secretion from bacteria and transport to host cells. The mitochondrial targeting signal (residues 1–24) and the nucleolar targeting domain (residues 21–74) enable EspF to target the mitochondria and nucleolus of the host cell. The C-terminal region comprises 3–4 proline-rich repeats (PRRs) (Figure 1A). The highly conserved RxAPxxP motif can bind specifically with the SH3 binding domain of the host cell SNX9 protein. The EspF protein can also bind to the N-WASP protein through the xHLAAYExSKxxxx sequence (Hua et al., 2018a).

It has been reported that EspF is involved in the TJ disruption process. EPEC lacking *espF* is unable to disrupt TER and distribute occludin from the TJ (Mcnamara et al., 2001). EspF protein can also promote the endocytosis of Crumbs3 and disrupt epithelial cell polarity, change the absorption of ions and solutes by membrane transporters, and promote EPEC-associated diarrhea (Tapia et al., 2017b). In addition, EspF protein can manipulate TJ through interaction with host proteins. EspF binds to N-WASP, ARP2/3, and scaffold protein ZO-1, inducing actin polymerization and depolymerization, thus promoting the pedestal formation hereafter and resulting in the imbalance of actin polymerization-depolymerization cycles (Peralta-Ramirez et al., 2008). The depolymerization of actin destructs TJ through caveolin-mediated endocytosis of occludin (Shen and Turner, 2005). EspF may combine with Calmodulin through 14-3-3ζ to activate and phosphorylate MLC, thereby disturbing the TJ barrier process (Hua et al., 2018a). A previous study revealed that EspF's binding to SNX9 could reorganize the actin pedestal, recruit active aPKC to actin at cell-cell borders, and promote endocytosis of occludin, thus destabilizing polarity complexes, which ultimately results in TJ perturbation (Weflen et al., 2009).

Despite the extensive evidence implicating EspF in the disruption of TJ barrier structure and function, a detailed molecular mechanism has not yet been elucidated. Our previous study demonstrated that host protein-Annexin A6 (ANXA6) co-precipitated with EspF (Hua et al., 2018b). ANXA6 is a scaffold protein that links the membrane microdomains with the cell cytoskeleton organization (Grewal et al., 2017). Here, we applied fluorescence resonance energy transfer (FRET) and co-immunoprecipitation (CO-IP) to further confirm the interaction between EspF and ANXA6 and validate the critical binding domain of their interaction. Our FRET and CO-IP analyses confirmed that EspF binds to ANXA6 through its C-terminal domain. In addition, we examined the effect of EspF-ANXA6 on TJs with a particular focus on MLCK, which phosphorylates several targets crucial for the contraction of actin and TJ breakdown. The data presented herein suggest that EspF-ANXA6 may contribute to TJ disruption through the MLCK-MLC pathway.

## Results

### EspF-ANXA6 Protein Complex Produces the FRET Phenomenon in Cells

**Figure 2:**
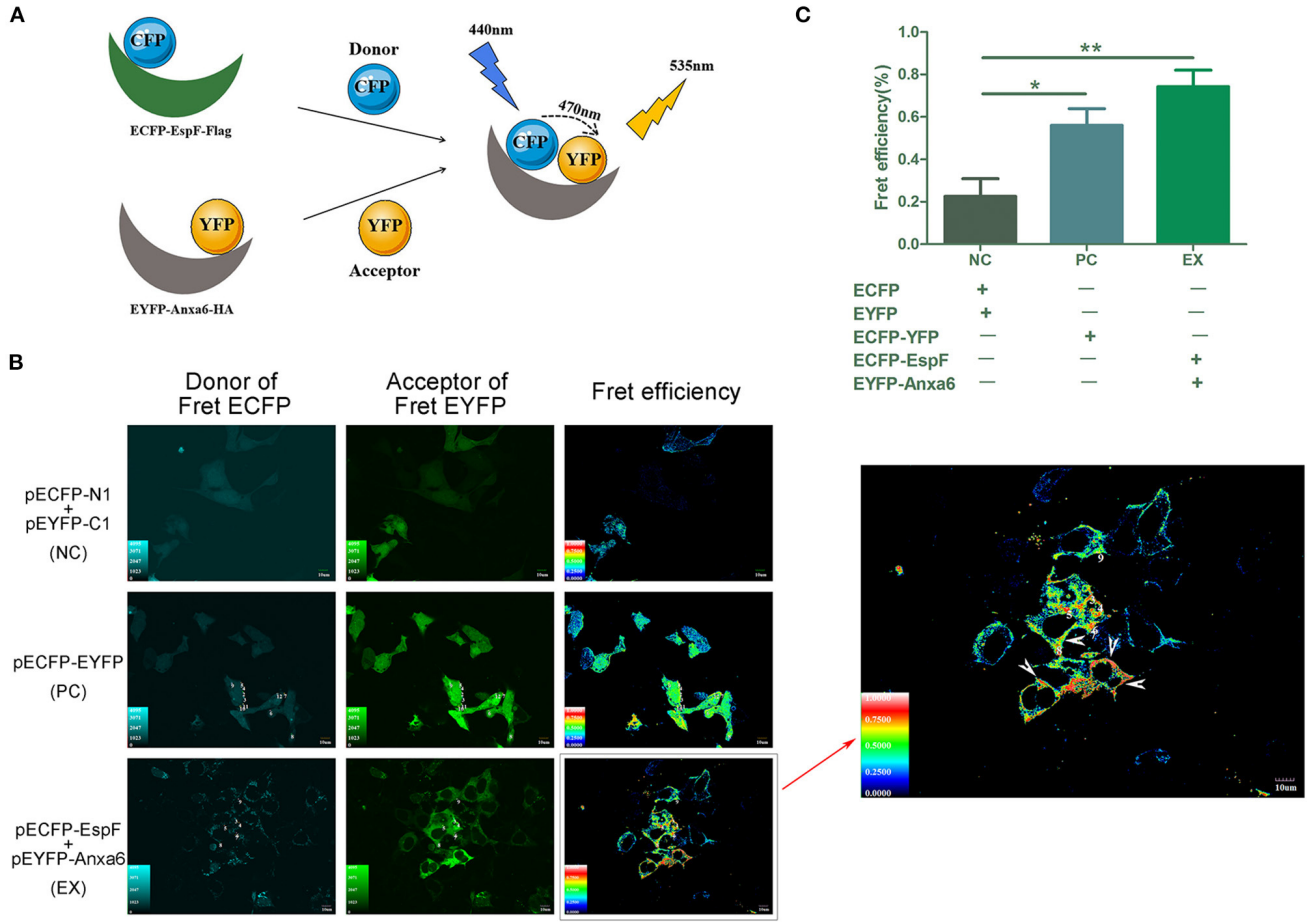
Analyses of the interaction between EspF and ANXA6 protein by FRET. Principle of the FRET assay was shown **(A)**. *espF* was fused to the N-terminal of pECFP-N1, *anxa6* was fused to the C-terminal of pEYFP-C1. CFP acts as the donor, YFP acts as the acceptor. A FRET phenomenon occurs when the distance between the two proteins is lower than 10 nm, indicating an interaction between these two proteins. EspF-ANXA6 protein complex produces FRET phenomenon in HEK293 cells **(A)**. Cells with FRET phenomenon are indicated by the arrow **(B)**. Cells co-transfected with pECFP-N1 and pEYFP-C1 were set as negative control (NC). Cells transfected with pECFP-EYFP, in which distance between ECFP and EYFP is below 10 nm and produce a FRET phenomenon, were positive control (PC). Cells co-transfected with pECFP-EspF and pEYFP-Anxa6 were set as the experimental group (EX). Quantitative analysis of FRET efficiency between each group is shown **(C)**. Error bars represent the means ± SD of triplicates. *T*-test results are between the EX/PC and NC, *Statistically significant difference. *p < 0.05. **p < 0.01.

We exploited FRET to visualize the interaction between EspF and ANXA6. Mammalian expression plasmids encoding EspF fused to the N-terminal of a cyan fluorescent protein with the flag tag (pECFP-EspF-flag, CE), and mammalian expression plasmids encoding ANXA6 fused to the C-terminal of yellow fluorescent protein (pEYFP-ANXA6-HA, YA) (Figure 1B). We investigated the function of the EspF-ANXA6 complex by co-expressing CE and YA in HEK293 cells. ECFP served as a FRET donor and EYFP as the acceptor; the emission spectrum of ECFP overlaps with the absorption spectrum of EYFP. When the fluorescence emitted by the donor overlaps with the absorption spectrum of the acceptor chromophore, and the distance between the two proteins is <10 nm, a FRET phenomenon occurs (Algar et al., 2019), indicating an interaction between the two proteins (Figure 2A).

HEK293 cell lines co-transfected with pECFP-N1 and pEYFP-C1 were set as a negative control (NC). Cells transfected with pECFP-YFP were a positive control (PC). Cells co-transfected with CE and YA were the experimental group (EX). The FRET efficiency of each group was assessed by fluorescence microscopy. The results showed that the average FRET efficiency of NC was 22.6, 56% for PC, and 74% for the experimental group. As shown in Figure 2, positive FRET signals were observed in positive control cells and cells co-expressing EspF and ANXA6, yielding significantly higher FRET efficiency than negative control cells. These findings indicate that EspF and ANXA6 can form a protein complex in cells. As shown by the arrow, FRET is mainly displayed in places close to the inner cell membrane (Figure 2B), indicating that these two proteins mostly interact at the inner cell membrane; this result is consistent with a study that described the ANXA6 protein as an annexin protein (Cornely et al., 2011).

### EspF Protein Interacts With Host ANXA6 Protein Through Its C-Terminal Domain

**Figure 3:**
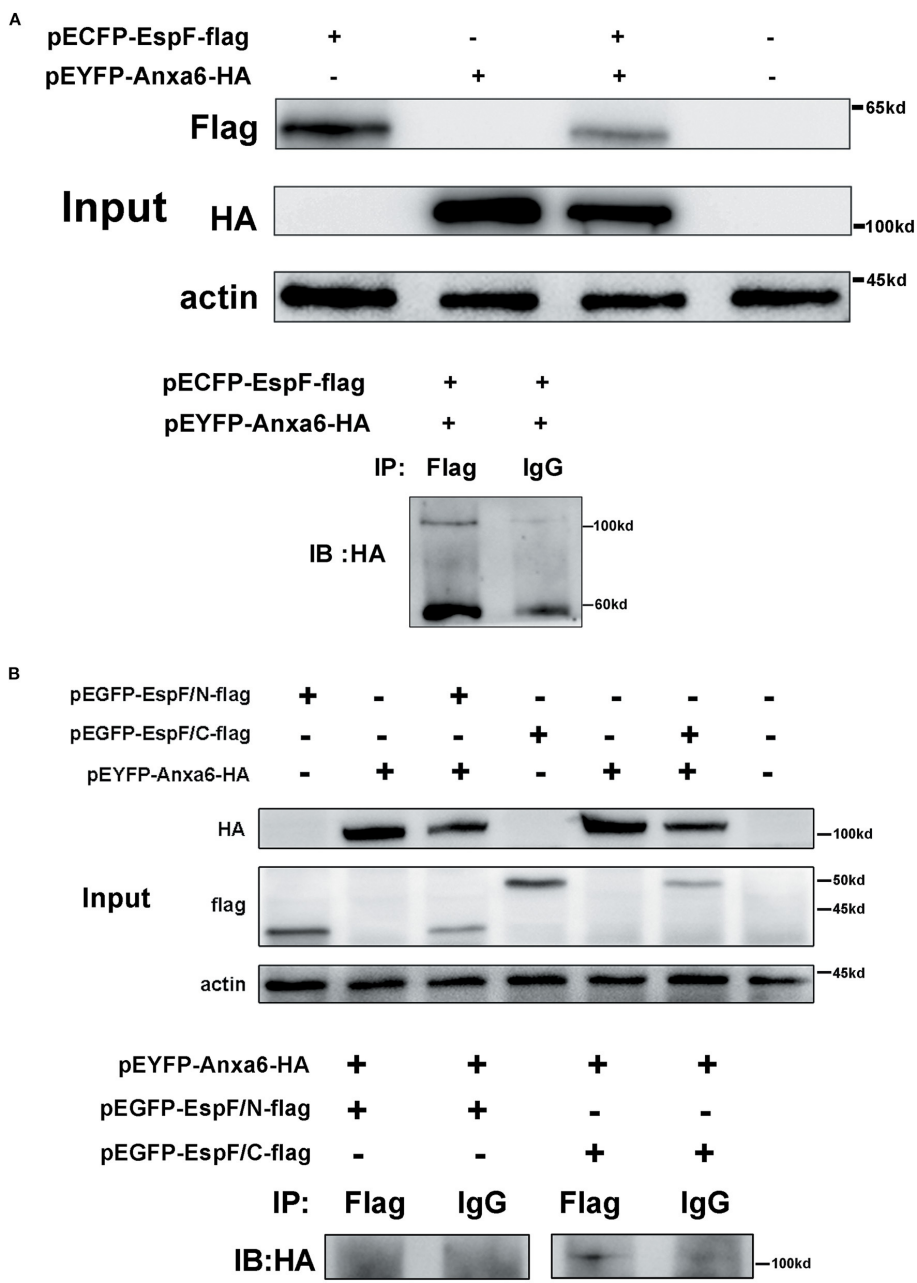
CO-IP confirms the interaction between EspF protein and ANXA6 protein **(A)** and the specific protein domain of EspF that interacts with ANXA6 **(B)**. Lysates of HEK293 cells over-expressing EspF or ANXA6, or co-overexpressing EspF and ANXA6, or co-overexpressing EspF/N or EspF/C and ANXA6 were detected by SDS-PAGE and western blotting (Input). The indicated interacting proteins were immunoprecipitated with the indicated antibodies (IB); normal mouse IgG was used as a negative control.

We employed the traditional CO-IP method to further validate the specific interaction domain of EspF with ANXA6. First, pECFP-EspF-Flag and pEYFP-ANXA6-HA were co- and single-transfected into HEK293 cells, and then the HEK293 cell lysate was obtained. We observed that EspF-Flag co-precipitated with ANXA6-HA. In contrast, interactions were not detected between IgG and ANXA6-HA (Figure 3A). Thus, we confirmed the direct binding of ANXA6 with the recombinant full-length EspF-Flag protein.

The N-terminal domain of the EspF protein plays a decisive role in EspF's targeting to mitochondria and nucleoli of host cells. In contrast, the C-terminal domain contains the primary protein-binding site with SNX9 and N-WASP (Hua et al., 2018a) (Figure 1A). It remains unknown which domain acts in the binding to ANXA6. Thus, we applied CO-IP to determine the specific EspF domain that contributes to the binding activity to ANXA6. We cloned the N-terminal (1–73 aa) and C-terminal (74–248 aa) sequences of EspF protein into the eukaryotic expression vector pEGFP-N1 and carried flag tag, respectively (Figure 1B). Then, we conducted co-transfection. Notably, as shown in Figure 3B, EspF C-terminal protein retained binding activity with ANXA6, whereas the N-terminal protein lost the ability to bind to ANXA6.

These findings suggest that EspF targets the host ANXA6 protein through its C-terminal. The biological function of EspF's binding to host ANXA6 protein and the role of EspF-ANXA6 in EHEC pathogenicity remains to be elucidated.

### Constitutive Expression of EspF and ANXA6 Protein Decreases the Level of ZO-1 and Occludin and Disrupts the Distribution of ZO-1

**Figure 4:**
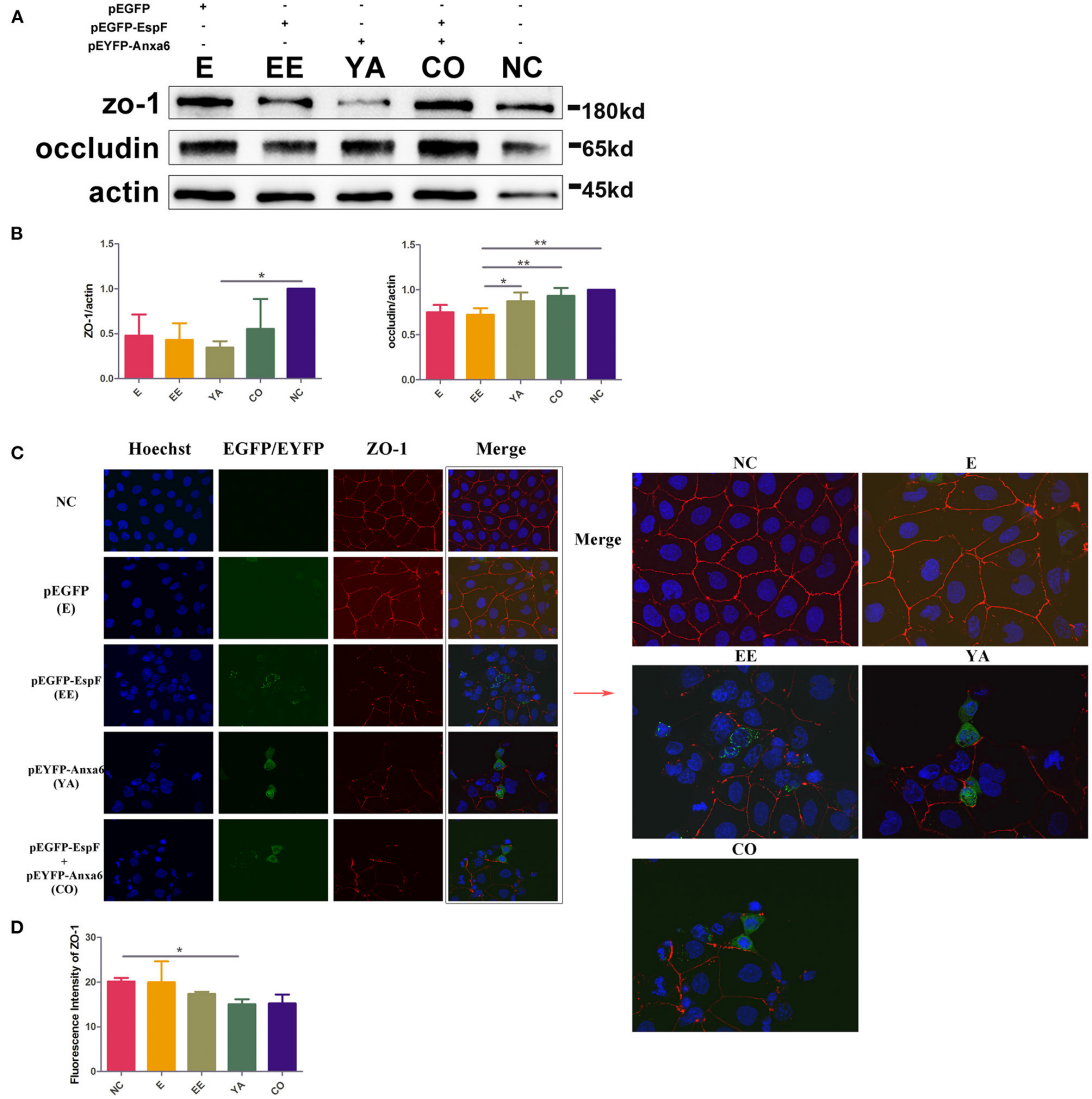
Plasmid-encoded EspF and ANXA6 proteins decrease the expression levels of ZO-1 and occludin and disrupt the distribution of ZO-1 in transfected Caco-2 cells. **(A,B)** The expression levels and grayscale analysis of WB strips in Caco-2 cells transfected with plasmids as indicated. The grayscale analysis was performed by ImageJ software. Error bars represent means ± SD from three independent experiments. **(C)** Immunofluorescence microscopy of ZO-1 protein. The nucleus was labeled with Hoechst in blue. ZO-1 was labeled with anti-ZO-1 antibody in red. Green indicates fluorescence expressed by the fluorescent plasmid. Images were acquired on an FV1000 confocal laser scanning microscope using a 60X oil objective. **(D)** Quantification analysis of fluorescence intensity of ZO-1 by ImageJ software. Error bars represent means ± SD from three independent experiments. NC, non-transfected cells; E, cells transfected with Pegfp; EE, cells transfected with pEGFP-EspF; YA, cells transfected with pEYFP-Anxa6; CO, cells co-transfected with pEGFP-EspF and pEYFP-Anxa6. *Statistically significant difference. *p < 0.05. **p < 0.01.

We detected the expression level of TJ proteins ZO-1 and occludin in Caco-2 cells. Plasmids pEGFP-EspF-Flag (EE) and pEYFP-ANXA6-HA (YA) were single- and co-transfected (CO) into Caco-2 cells. Cells transfected with plasmids pEGFP-N1 (E) were set as a blank control. The constitutive expression of EspF (EE) and ANXA6 (YA) depleted ZO-1 and occludin proteins. The co-transfected group decreased the level of ZO-1, but did not affect occludin. Meanwhile, the reduction effect on occludin protein was not as obvious as that on ZO-1 protein. Of the two proteins, ANXA6 appeared to have a stronger ability to reduce the level of ZO-1, while EspF was more efficient in depleting occludin (Figures 4A,B).

Between the monolayer Caco-2 cells, an “upper-like” complete and dense structure was formed, that is, TJs. The TJ protein ZO-1 is continuously distributed along the membrane under normal conditions. We transfected plasmids encoding EspF and ANXA6 proteins, and then observed the distribution of ZO-1 by immunofluorescence. As shown in Figure 4C, in non-transfected Caco-2 cells (NC), ZO-1 protein was continuously distributed along the cell membrane, with a complete structure and clear boundaries. In the E group, the fluorescence of ZO-1 appeared mostly continuous. Compared with NC, the single-transfected with EspF (EE) or ANXA6 (YA), and their co-transfected (CO) groups showed looser, discontinuous, and weakened fluorescence signals distributed in a zigzag pattern, with clear gaps and crack-like appearance. The fluorescence intensity of ZO-1 was quantified by ImageJ software, as shown in Figure 4D. The signal intensity of ZO-1 in the EE, YA, and CO groups were lower than in non-transfected group. The intensity of ZO-1 in the YA group was significantly reduced compared to the non-transfected group. These results indicate that ANXA6 protein has a stronger ability than EspF protein to facilitate the collapse of the essential cellular structure ZO-1.

### Co-expression of EspF and ANXA6 Disrupts TJ Integrity and Declines the Level of ZO-1 and Occludin

The T2A peptide is one of the most popular strategies used to express multiple genes in eukaryotic cells (Ahier and Jarriault, 2014). Co-transfection has some limitations, such as different transfection efficiency in different vectors, which results in an inconsistent expression level of fused protein. To determine the effects of the EspF-ANXA6 interaction on TJ destruction, we used the T2A peptide to construct an eukaryotic expression vector pEYFP-EspF-T2A-ANXA6-HA (EYEA) (Figure 1B), which enabled *espF* and *anxa6* to be expressed in equivalent amounts in the same cells.

**Figure 5:**
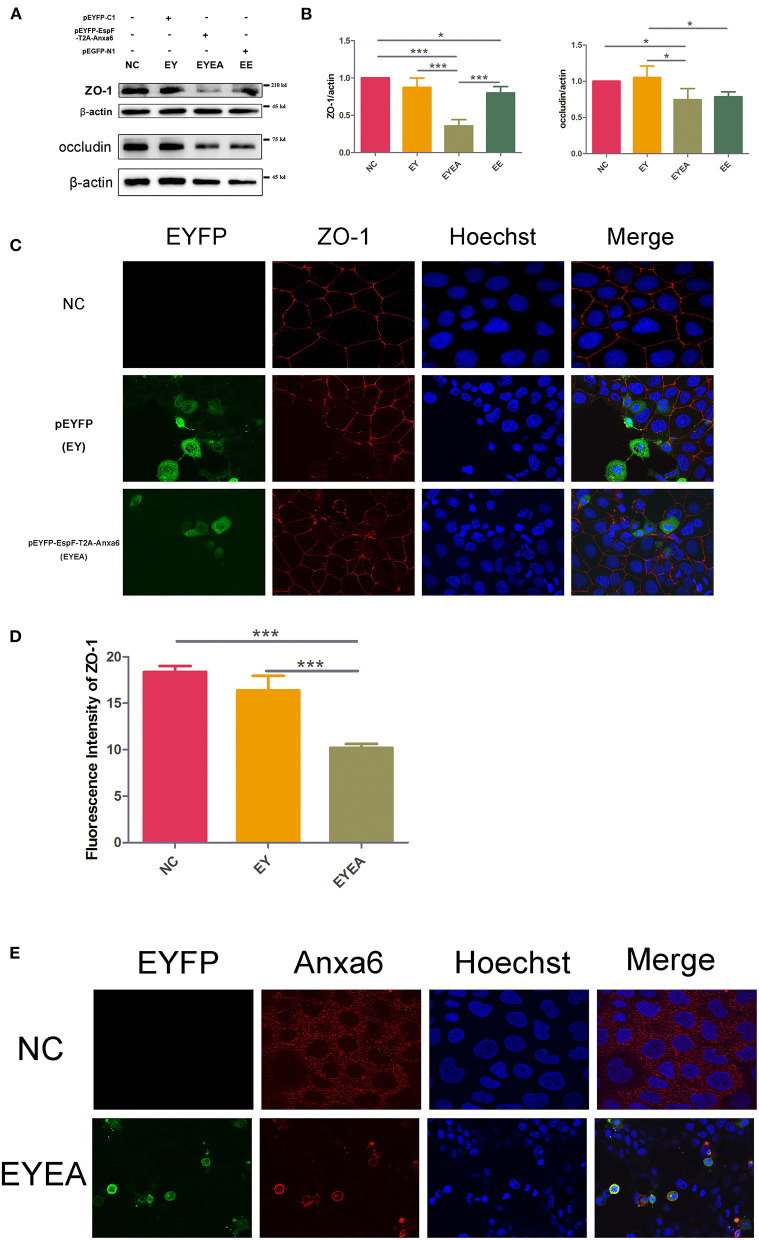
The co-expression of EspF and ANXA6 protein in Caco-2 cells reduced the level of ZO-1 and occludin and disrupted the structure of ZO-1. **(A,B)** The expression levels and grayscale analysis of WB strips in Caco-2 cells transfected with plasmids as indicated. The grayscale analysis was performed in ImageJ software. Error bars represent means ± SD from three independent experiments. **(C,D)** Immunofluorescence and quantification analysis of ZO-1 protein. Nucleus was labeled with Hoechst in blue. ZO-1 was labeled with anti-ZO-1 antibody in red. Green indicates fluorescence expressed by the fluorescent plasmid. Images were acquired on an FV1000 confocal laser scanning microscope using a 60X oil objective. The fluorescence intensity of ZO-1 was analyzed by ImageJ software. Error bars represent means ± SD from three independent experiments. *Statistically significant difference. *p < 0.05. ***p < 0.001. **(E)** Immunofluorescence of ANXA6 protein in Caco-2 cells. As described in **(C,D)** ANXA6 was labeled with an anti-ANXA6 antibody in red. Images were acquired on an FV1000 confocal laser scanning microscope using a 60X oil objective. NC, non-transfected cells; EYEA, cells transfected with pEYFP-EspF-T2A-ANXA6.

First, we evaluated the level of ZO-1 and occludin in Caco-2 cells that expressed EspF-ANXA6. Compared with NC, the levels of ZO-1 and occludin in the EYEA group were significantly reduced. The reduction effect on ZO-1 was more significant than that on occludin. In addition, the level of ZO-1 and occludin in cells expressing plasmid-encoded EspF (EE) was also lower than in the NC group (Figures 5A,B).

We then observed the localization of ZO-1 in Caco-2 cells transfected with EYEA. Non-transfected Caco-2 cells (NC) and cells expressing EYFP (EY) were used as controls. As shown in Figure 5C, ZO-1 was typically localized at mature TJ in NC. In EY, ZO-1 staining appeared marginally reduced in cells that expressed EYFP and normal in other cells. ZO-1 was markedly discontinuous and reduced at TJ in EspF-ANXA6-expressing cell lines. Consistently, the fluorescence intensity of ZO-1 in the EYEA group was significantly lower compared with the NC and EY groups (Figure 5D). These results showed that the ectopically expressed EspF-ANXA6 disrupted TJ integrity. In addition, the cellular localization of the ANXA6 protein was detected by specific antibodies. Interestingly, the results revealed that endogenous ANXA6 protein was mainly distributed in cytoplasm. While cells transfected with EYEA, ANXA6 protein was predominantly accumulated in the cell membrane, which is consistent with its annexin properties (Figure 5E). Remarkably, cells expressing the EspF-ANXA6 proteins showed significant nuclear shrinkage, but the mechanism behind this phenotype remains unknown.

The data presented herein support the notion that EspF protein promotes the disruption of TJ integrity and accelerates the depletion of TJ proteins through the interaction with the host ANXA6 protein.

### EspF-ANXA6 May Perturb TJ Function Through the Activation of the MLCK-MLC Pathway

**Figure 6:**
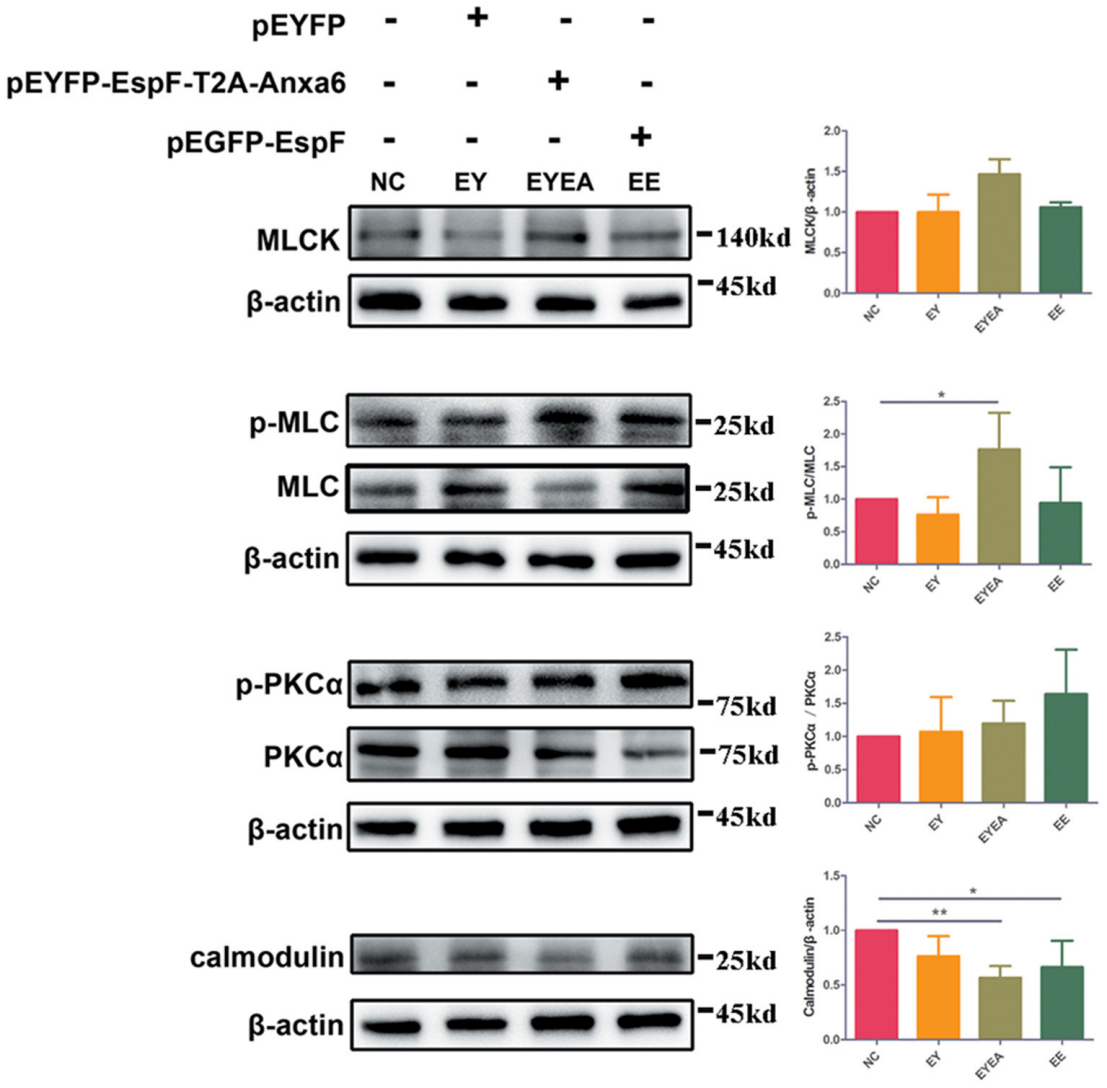
The co-expression of EspF and ANXA6 protein in Caco-2 cells activates the MLCK-MLC pathway. The WB strips of MLCK-MLC pathway proteins in different plasmid-transfection groups, as indicated, are shown. Increased expression of MLCK, phosphorylated expression of MLC and PKCα, and decreased expression of Calmodulin in the EspF-ANXA6 transfected group, suggesting that EspF-ANXA6 activates the MLCK-MLC pathway, which may perturb TJ function. NC, non-transfected cells; EY, cells transfected with pEYFP; EYEA, cells transfected with pEYFP-EspF-T2A-ANXA6; EE, cells transfected with pEGFP-EspF. *Statistically significant difference. *p < 0.05. **p < 0.01.

MLC phosphorylation has been reported in EPEC infection. Increased levels of MLCK can cause MLC phosphorylation, while the inhibition of MLCK was reported to attenuate bacterial-induced epithelial damage both *in vivo* and *in vitro* (Conlin et al., 2009). Our results in HEK293 cells (data not shown) demonstrated that ANXA6 could cause phosphorylation of PKCα, which is a crucial pathway protein of PKC and MLCK signaling. We hypothesized that EspF might be involved in the regulation of the TJ signaling pathway by binding with ANXA6 protein. To determine the relevance of EspF-ANXA6 interaction for TJ destruction, we performed western blot analysis with the MLCK-MLC signaling pathway-related proteins in Caco-2 cells. As shown in Figure 6, the level of MLCK protein was higher in cells expressing EspF-ANXA6 than in non-transfected (NC), EY, and EE groups. This result suggests that MLCK was activated by the EspF-ANXA6 interaction but not by EspF alone. Similar results were obtained by determining the amount of phosphorylated MLC (p-MLC). The amount of p-MLC increased as a function of the activity of ANXA6, which was higher in cells expressing EspF-ANXA6 than in cells expressing only EspF. In addition, the level of p-MLC was significantly increased compared with the NC group. The amount of phosphorylated PKCα (p-PKCα) was higher in cells expressing EspF than in cells expressing EspF-ANXA6, both of which were higher compared with the NC group. This result indicates that EspF protein alone can cause phosphorylation of PKCα. As MLCK is a highly specialized Calcium/Calmodulin-dependent kinase (Behanna et al., 2006), we also examined the expression level of Calmodulin protein. The level of Calmodulin in cells expressing EspF-ANXA6 and cells expressing EspF was significantly lower compared with non-transfected cells. However, the amount of Calmodulin was higher in the EE group than in the EYEA group, suggesting that the interaction with ANXA6 helps EspF protein to decrease the expression of Calmodulin. Taken together, these results suggest a mechanism by which the EspF-ANXA6 interaction perturbs TJ function through the MLCK-MLC pathway.

### EspF Protein Is Critical for EHEC Infection to Decrease TER, and Down-Regulates the Expression Level of ZO-1 and Occludin

**Figure 7:**
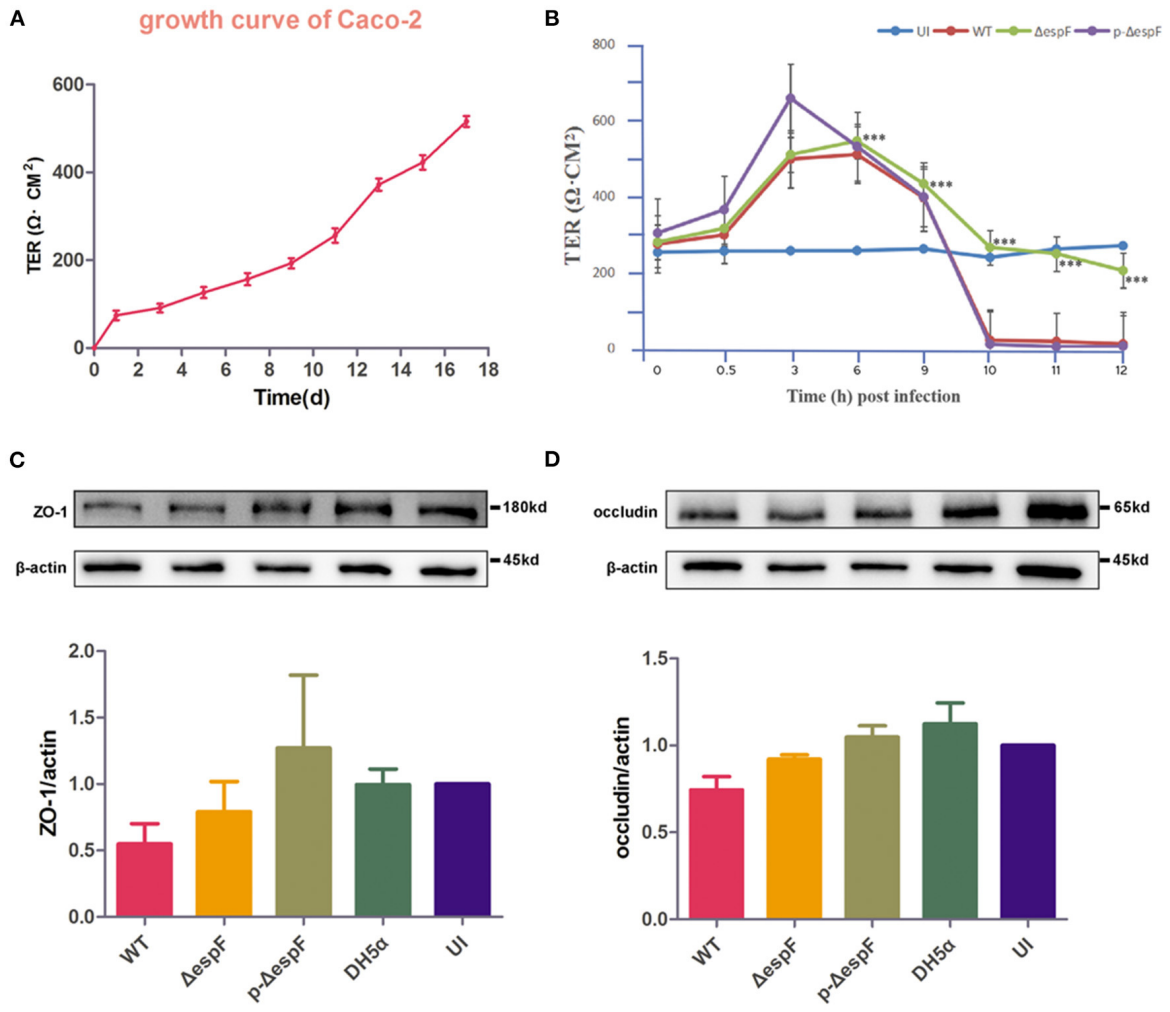
Schematic diagram of changes in TER and expression level of ZO-1 and occludin in Caco-2 cells infected with EHEC O157:H7. **(A)** TER growth curve of Caco-2 cells. **(B)** The curve of TER value in Caco-2 cells infected with EHEC EDL933w strains (WT), *espF*-deficient strains (Δ*espF*), and *espF*-complementation Strains (p-Δ*espF*). Data are presented as means ± SD from three independent experiments. *Statistically significant difference. ***p < 0.001. **(C,D)** Schematic diagram of TJ protein expression levels and grayscale analysis of WB strips in Caco-2 cells infected with WT, Δ*espF*, p-Δ*espF*, and DH5α. The grayscale analysis was performed by ImageJ software. Error bars represent means ± SD from three independent experiments. UI, uninfected cells.

TER, which can reflect the barrier function of IECs, is commonly used to detect cell permeability. Several investigators have reported that EPEC plays a vital role in TER loss (Tapia et al., 2017a, 2020). To determine whether EHEC infection alters the intestinal barrier function and to explore the role of EspF in this process, we first examined the TER value during the growth of Caco-2 cells. From the growth curve of Caco-2 cells, the TER value increased with the extension of the culture time and increased significantly from day 11 to day 13, indicating that the cells entered the rapid growth phase around day 11. By day 18, the TER value gradually stabilized, and a monolayer of cells was gradually formed (Figure 7A).

Caco-2 cells were seeded on permeable Transwell filters. We infected the Caco-2 cells, which reached the rapid growth phase with the EHEC EDL933w strain (WT), *espF*-deficient strain (Δ*espF*), and *espF*- complementation strain (p-Δ*espF*) (MOI = 100), and monitored TER over time. The TER value of uninfected (UI) group was stable. Six hours after infection, the TER value of the experimental group began to decline, and we observed a dramatic loss of TER in monolayers infected with WT at the 10th hour. In contrast, the Δ*espF* group eased the decline, whereas the TER value of the p-Δ*espF* group dropped sharply at the 10th hour, similar to the WT group (Figure 7B). These data indicate that EHEC infection causes EspF-related TER decline.

Since the TJ constituent proteins occludin and ZO-1 have been linked to the regulation of TJ permeability, we examined the expression level of ZO-1 and occludin in cell lysates derived from Caco-2 cells infected with WT, Δ *espF*, and p-Δ *espF*. Besides the UI group, we also set DH5α as a control group. Compared with the UI group, the level of ZO-1 and occludin decreased in the WT and Δ *espF* groups. Compared with the WT group, the level of ZO-1 and occludin was elevated in the Δ *espF* group (Figures 7C,D). No reduction was observed in the p-Δ *espF* group, which was expected to have a similar tendency to the WT group.

These data indicate that EHEC O157:H7 infection not only induces TER loss in Caco-2 cells but also decreases the expression level of TJ proteins ZO-1 and occludin, both of which depend on the effector protein EspF.

## Discussion

EspF has been reported to disrupt host intestinal barrier function. However, most related data are from research on EPEC, which is reported to be more efficient at barrier function alteration than EHEC (Viswanathan et al., 2004). As TJ has been suggested to be an important facet of bacterial pathogenesis, the mechanism of EHEC-mediated TJ disruption and the effect of EspF on TJ breakdown in EHEC deserves to be investigated.

The *espF* gene has an 87% sequence similarity between EPEC and EHEC (Hua et al., 2018a). Consistent with EPEC, we found that the *espF*-deficient strains lost the ability to sharply decrease TER in Caco-2 cells, indicating that EspF is required for EHEC-induced TER loss. Additionally, we found that the *espF*-deficient strains reduced the level of ZO-1 and occludin when compared with the non-infected group. However, the reduction effect was relatively alleviated compared with the WT group. These results demonstrated that in addition to EspF, some other proteins or factors reduce the level of tight junction proteins. Anand et al. reported that the EPEC effector Map is involved in the TJ disruption process through a mechanism that is distinct from that of EspF (Singh et al., 2018). Thus, TJ barrier disruption may be a result of multiple factors.

As EspF is involved in TER disruption, we hypothesized that EspF would interact with a specific host protein to interfere with the pathogenic phenotype. In our previous study, we employed the bimolecular fluorescence complementation (BiFC) method to examine the interplay between EspF and host proteins, which provided specific candidates that interact with EspF (Hua et al., 2018b). Among these candidates, ANXA6 showed a strong positive reaction with EspF. In this study, we used FRET and CO-IP to further validate the interaction between EspF protein and ANXA6 protein. FRET is an assay that detects the distance of two molecules within 10 nm (Algar et al., 2019); the fluorescence signals captured by a confocal microscope measure FRET efficiency and indicate the strength of protein-protein interactions. The combination of FRET and BiFC is widely used for visualization and identification of protein interactions in living cells (Kerppola, 2008; Shyu et al., 2008). We employed BiFC to identify host proteins that bind to EspF, and then applied FRET and CO-IP to validate the interactions, thus proofing the interaction between EspF and ANXA6.

Studies have shown that the cooperation of EspF with host proteins results in disruption of multiple functions of the TJ. For example, the EspF of rabbit EPEC (E22) binds to actin and profilin, which may enable EspF to regulate actin polymerization, thereby interacting with ZO-1 and ZO-2 and resulting in TJ protein redistribution. EspF protein also cooperates with N-WASP and ARP2/3, inducing actin nucleation and disrupting paracellular permeability (Peralta-Ramirez et al., 2008). EPEC EspF collaborates with cytokeratin 18 and 14-3-3ζ, resulting in the alteration of the intermediate filament network (Viswanathan et al., 2004). Before our study (Hua et al., 2018b), there were no reports of EspF-ANXA6 protein interaction. We found that EspF binds to ANXA6 protein through its C-terminal. Interestingly, mitochondrial dysfunction, caused by the EspF N-terminal domain, is not involved in TJ barrier disruption (Viswanathan et al., 2008). EPEC EspF was found to induce multinucleation and cell fusion events, which rely on its C-terminal domain (Dean and Kenny, 2013). The function of the EspF C-terminal domain may be underestimated, as we do not know if the PRR repeats are simply a result of DNA replication or have a host-specific function. EPEC EspF was reported to bind SNX9 and N-WASP proteins owing to the SH3 (Src homology-3) and CRIB (Cdc42/rac interactive binding) motifs of the C-terminal; their interactions may disrupt TJ through an endocytic hijacking process (Garber et al., 2018). The C-terminal domain of EspF protein appears to act as a scaffold to link EspF with other proteins and then exert biological functions. However, the specific ANXA6-binding site remains to be further investigated.

ANXA6 is a Calcium/Calmodulin-dependent phospholipid-binding protein and is implicated in various cellular processes (Grewal et al., 2017). ANXA6 was reported to interact with Influenza A virus M2 protein to impair the virus infection (Ma et al., 2012). Additionally, ANXA6 can bind to actin and remodel the actin cytoskeleton by forming membrane-cytoskeletal complexes (Hayes et al., 2004). The significance of the actin cytoskeleton for TJ is well-established (Ivanov et al., 2010). The involvement of ANXA6 in the endocytosis process (Enrich et al., 2017) is intriguing as the TJ is regulated by actin as well as endocytosis (Shen and Turner, 2005).

We detected the effect of EspF-ANXA6 on TJs. Both constitutive expressions of EspF and ANXA6 could decrease the level of TJ proteins. Compared with EspF, ANXA6 had a stronger ability to reduce the level of ZO-1 but a weaker ability to reduce the level of occludin. The co-transfection of EspF and ANXA6 did not reduce the TJ proteins as well as the single transfection of EspF or ANXA6. We speculate that there may be competition between co-transfection plasmids, leading to differences in transfection efficiency and protein expression level. The plasmid-encoded EspF and ANXA6 could also disrupt the distribution of ZO-1. Notably, we found that ANXA6 can induce PKCα protein phosphorylation in HEK293 cells. In Caco-2 cells, EspF could induce the phosphorylation of PKCα protein, but the interaction with ANXA6 seemed to weaken this ability of the EspF protein. PKCα has been reported to be activated and recruited to form adhesion pedestals upon EHEC/EPEC infection. PKCα also plays a role in the formation of the adhesion base and regulation of the cytoskeleton (Crane and Oh, 1997; Shen-Tu et al., 2014), but the precise mechanism leading to these phenotypes remains undetermined.

The TJ is regulated by multiple signaling pathways, such as PKC, PKA, MLCK, and MAPK (Gonzalez-Mariscal et al., 2008). Among these, the PKC and MLCK signaling pathways have a cross-action in regulating TJs. PKC can activate MLCK and induce the phosphorylation of MLC, which may lead to actin contraction and cytoskeletal remodeling, resulting in TJ destruction (Turner et al., 1999; Banan et al., 2003). The activation of MLCK can trigger TJ endocytosis and regulate the TJ barrier by mediating actin cytoskeleton reorganization (Shen, 2012). EPEC infection was reported to cause MLCK activation (Yuhan et al., 1997). The involvement of MLCK and PKC in the EHEC-induced decrease in the barrier function in T84 cells has previously been reported (Philpott et al., 1998). In this study, we found that EspF-ANXA6 can activate MLCK, induce the phosphorylation of MLC and PKCα, and decrease the expression level of Calmodulin protein. The data highlight that EspF activation of the MLCK-MLC pathway via binding ANXA6 is essential for the breakdown of TJs.

There are some limitations to this study. First, the complementation strain of *espF* does not have the same ability to decrease ZO-1 and occludin as the wild-type strain. The *espF* gene was cloned into pBAD33 plasmid to construct the complementation strain (Hua et al., 2015). There may have been two reasons why the complementation strain did not work. One is that the *espF* gene was not complemented to the original position in the bacterial genome in the locus of the enterocyte effacement (LEE) pathogenicity island (Holmes et al., 2010). The other reason is that due to the pBAD expression system, the expression level of EspF was controlled by the regulation of arabinose, which caused the instability. The second primary limitation to this work is that the specific sites of EspF's binding to ANXA6 remain to be determined. In addition, endogenous ANXA6 protein in Caco-2 cells does not exert its function on barrier integrity and is mainly distributed in the cytoplasm. The exogenous expression of ANXA6 protein is distributed in the inner cell membrane. The reason for this phenotypic change is not presently understood. Further research and an *in vivo* model are needed to verify the specific binding sites and to understand the mechanism of EspF-ANXA6 in the regulation of TJ.

**Figure 8:**
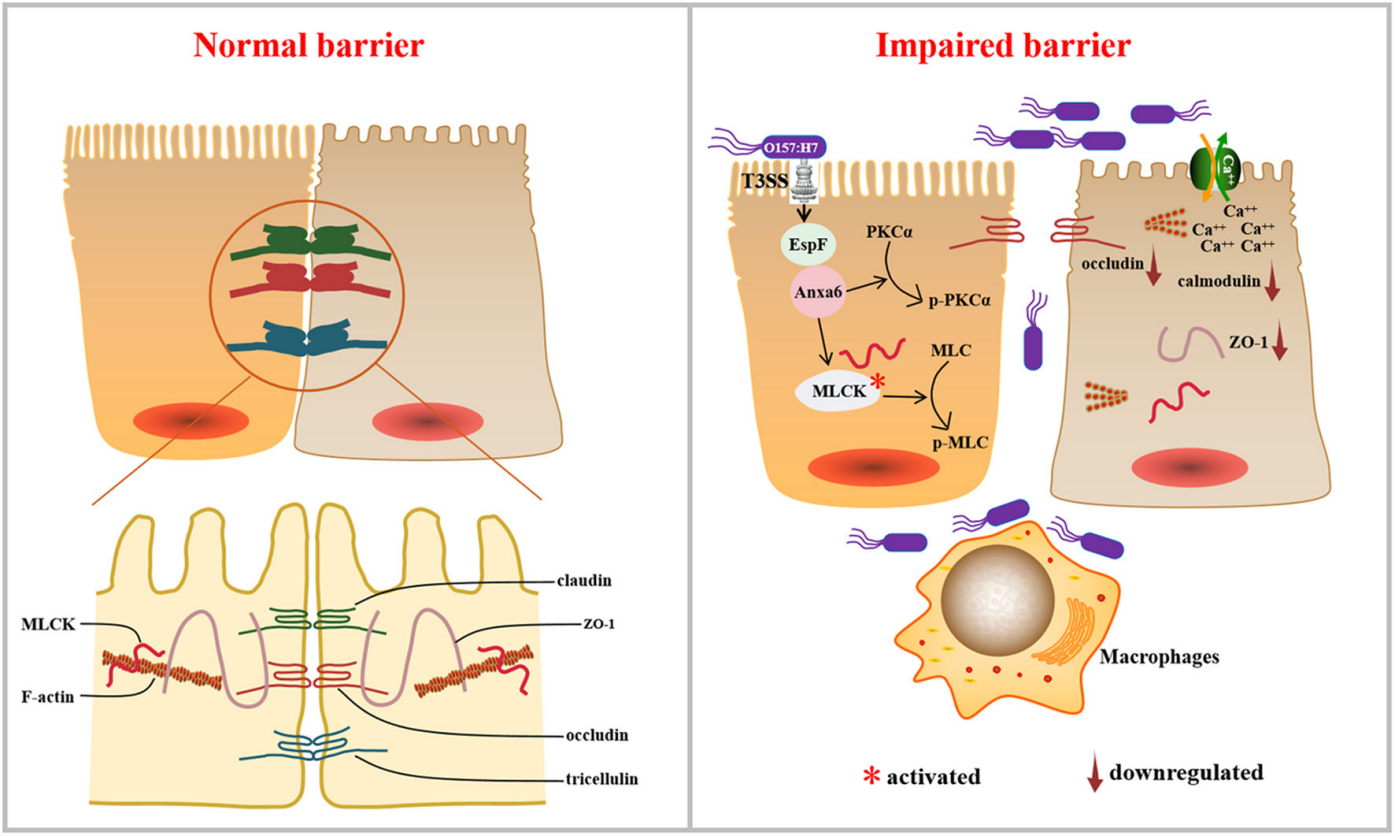
Working model: the pathogen EHEC disrupts TJs during infection, which depends on its effector protein EspF. EspF interacts with host protein ANXA6, depletes the expression levels of ZO-1 and occludin, disrupts the distribution of ZO-1, activates MLCK, induces the phosphorylation of MLC and PKCα, and decreases the expression of Calmodulin, which results in the dysregulation of the TJ.

TJ's dysregulation could lead to some effects, such as impairment of paracellular transport and increased permeability. These are crucial steps in EHEC pathogenesis and their deregulation could result in diarrhea and inflammation (Krug et al., 2014). Collectively, the results reported herein suggest a mechanism by which EspF disrupts intestinal epithelial TJs (Figure 8). The EHEC effector protein EspF interacts with a novel host protein ANXA6 through its C-terminal domain. The EspF-ANXA6 complex decreases and disrupts TJ proteins in Caco-2 cells, activates MLCK, induces MLC and PKCα phosphorylation, and decreases the level of Calmodulin protein. EspF, therefore, appears to destruct TJs via interaction with ANXA6 in an MLCK-MLC-dependent manner, thus contributing to the pathogenicity of EHEC.

## Materials and Methods

### Bacterial Strains and Cells

Enterohemorrhagic *E. coli* O157:H7 EDL 933 (WT), *espF*-deficient strain (Δ*espF*), *espF*-complementation strain (p-Δ*espF*), and DH5α were preserved in our laboratory. Strains were routinely grown overnight at 37°C in Luria-Bertani (LB) broth with 0.1% kanamycin (Δ*espF*) or 0.4% chloramphenicol, and 0.1% L-arabinose (p-Δ*espF*). Before infection, strains were cultured in DMEM (Dulbecco's Modified Eagle Medium, Gibco, CA, USA) with low glucose (1 g/L) to induce the expression of T3SS (Njoroge et al., 2012).

Caco-2 cells and HEK293 cells were routinely cultured in DMEM (4.5 g/L glucose) supplemented with 10% fetal bovine serum (FBS, Gibco) and 1 × penicillin-streptomycin solution (Gibco) under humidified 5% CO_2_ at 37°C.

### Plasmid Construction

The gene encoding EspF was derived from human EHEC O157:H7 EDL933 strain. The gene encoding ANXA6 was obtained (Wenbin Ma, Sun Yat-sen University, China) and preserved in our laboratory. These two genes were cloned in different vectors to generate a label-tagged *espF* and *anxa6* constructs (Figure 1B). Primer pairs C-F: CCCAAGCTTGCCACCATGCTTAATGGAATTAGTAACGCT and C-R: CGCGGATCCTGCTTATCGTCGTCATCCTTGTAATCCTTATCGTCGTCATCCTTGTAATCCTTATCGTCGTCATCCTTGTAATCCCCTTTCTTCGATTGCTC were used to construct the plasmid pECFP-EspF-flag; primers A-F: CCCAAGCTTCGATGGCCAAACCAGCACAGGG and A-R: CGGGGTACCTTAGGCATAATCGGGTACATCGTAAGGGTAGTCCTCACCACCACAGAG were used to construct the plasmid pEYFP-ANXA6 -HA. Plasmid pEGFP-EspF, pEGFP-EspF/C-flag, and pEGFP-EspF/N -flag were preserved in our laboratory (Hua et al., 2018b). Plasmid pECFP-N1, pEYFP-C1, and pECFP-YFP were obtained from professor Hongjuan Peng (School of Public Health, Southern Medical University, China).

To mimic the interaction of EspF protein and ANXA6 protein in cells, we used 2A self-cleaving peptide to construct a plasmid that can express EspF and ANXA6 in cells in equal amounts. Primers ET-F: CGGACTCAGATCTCGAGCTCAAGCTTCGATGCTTAATGGAATTAGTAA and ET-R: CCACGTCACCGCATGTTAGAAGACTTCCTCTGCCCT CCCCTTTCTTCGATTGCTC were used to amplify EspF-T2A, and primers AT-F: TCTAACATGCGGTGACGTGGAGGAGAATCCCGGCCCTAT GGCCAAACCAGCACAGGG and AT-R: CCGGTGGATCCCGGGCC CGCGGTACCTTAGGCATAATCGGGTACAT were used to amplify T2A-ANXA6-HA. Then the PCR products of these two fragments were cloned into the HindIII/KpnI restriction sites of pEYFP-C1 to generate pEYFP-EspF-T2A-ANXA6-HA using the ClonExpress method (MultiS One Step Cloning Kit, Vazyme, China). These constructs are listed in Figure 1B. All constructs were verified by DNA sequencing.

### TER Measurement

Caco-2 cells (2 × 10^5^) were seeded on 6.5 mm Transwell chambers with a pore size of 0.4 μm (Corning, NY, USA) and cultured for 10–14 days. Transepithelial resistance was measured every other day using a voltmeter (EVOM, FL, USA). The cell growth curve was drawn according to the TER value (Ω × cm^2^), and the rapid growth period of Caco-2 cells was determined. The cells were infected with WT, Δ*espF*, and p-Δ*espF* strains at their rapid growth period (multiplicity of infection of 100), and TER was measured three times for each well at the indicated time. The final TER values of each group were calculated by subtracting the resistance of the blank filter from the resistance of the filters containing cell lines and bacteria, then multiplied with the surface area of the 6.5 mm filters (0.33 cm^2^).

### Fluorescence Resonance Energy Transfer Assay

The day before transfection, HEK293 cells were seeded in glass-bottom cell culture dishes (Nest, China) with DMEM growth medium without antibiotics. When the cells were ~60 to 70% confluent, p-ECFP-EspF-flag (1 μg) and/or pEYFP-ANXA6-HA (1 μg) were transfected into cells by Lipofectamine 3000 transfection reagent (Invitrogen, Waltham, MA, USA). Cells co-transfected with pECFP-N1 and pEYFP-C1 were set as a negative control (NC). Cells transfected with pECFP-YFP were a positive control (PC) as the distance between the encoded protein CFP and YFP was <10 nm.

For the FRET assay, fluorescent images were captured 48 h after transfection using a confocal microscope (FV1000, Olympus, Japan). The samples were excited at 440 nm, and the emission detection wavelengths were 520 ± 20 nm. The fluorescence images were captured under an appropriate filter set. Regions of interest were selected, and background subtraction was completed for all images. FRET efficiency was obtained with the “series analysis tool” of the Olympus FluoView^TM^ FV1000 software and is shown as the mean of each experiment; each experiment was repeated at least three times.

### Co-immunoprecipitation Assays

HEK293 cells over-expressing EspF and/or ANXA6 were prepared, as mentioned previously in the FRET assay. Non-transfected and transfected monolayers were washed with cold PBS. The cells were lysed in immunoprecipitation assay buffer (RIPA, Beyotime, Shanghai, China) with 1 mM phenylmethanesulfonyl fluoride (PMSF, Bioss, Beijing, China) for 30 min at 4°C. The cell lysates were harvested with centrifugation at 13,000 g for 15 min at 4°C, and the supernatant was removed and kept on ice. Subsequently, cell lysates were incubated with the primary antibody (anti-flag and anti-HA) with gentle rotation at 4°C for 3 h. Then, ~25 μL protein A+G Agarose beads (Beyotime, Shanghai, China) were added and incubated with the immunoprecipitation reaction at 4°C overnight. The beads were washed three times with radioimmunoprecipitation assay buffer (RIPA buffer). The protein was eluted with SDS-PAGE loading buffer (CW027S, CWBIO, Beijing, China) and boiled for 10 min, then subjected to SDS-PAGE and western blot analysis. The blots were probed with antibodies against Flag (#F1804, mouse monoclonal, 1:1000, Sigma), HA (#AP0005M, mouse monoclonal, 1:1000, Bioworld), and actin (#66009-1-Ig, mouse monoclonal,1:6000, Proteintech).

### Immunofluorescence Assay

To examine the effect of plasmid-encoded EspF and ANXA6 on the distribution of ZO-1, we seeded Caco-2 cells in glass-bottom cell culture dishes and transfected cells with pEGFP-EspF (1 μg) or/and pEYFP-ANXA6-HA (1 μg); pEGFP-N1 was set as an empty plasmid control. After 48 h from transfection, cell monolayers were washed with ice-cold PBS and fixed with chilled methanol for 5 min at room temperature (RT), then blocked with 10% ready-to-use goat serum for 30 min at RT. The fixed cells were then stained using primary antibody diluted with 10% goat serum (#ab216880, rabbit polyclonal, anti-ZO-1, 1:500, Abcam) overnight at 4°C, followed by incubation with goat anti-rabbit secondary antibody (1:500, EarthOx, CA, USA) for 1 h. Then, the cells were stained for DNA using Hoechst (#C1027, Beyotime, Shanghai, China) for 10 min at RT. The samples were visualized on an FV1000 (Olympus, Japan) confocal laser scanning microscope. The images captured were grouped, projected, and analyzed with Olympus FluoView^TM^ FV1000 software.

Caco-2 cells were seeded and transfected with plasmid pEYFP-EspF-T2A-ANXA6-HA as described above, to detect the effect of interaction between EspF protein and ANXA6 protein on the distribution of ZO-1 and check the localization of ANXA6 protein. The primary antibody anti-ANXA6 (#720161, 1:500, Thermo Fisher Scientific, USA) was used to stain ANXA6. Then, cells were stained for DNA and visualized as described above.

### Western Blotting

The protein samples were loaded and separated by sodium dodecyl sulfate-polyacrylamide gel electrophoresis (SDS-PAGE). Electrophoresed proteins were transferred to polyvinylidene fluoride (PVDF) membranes (Merck Millipore, Darmstadt, Germany), then blocked with 5% bovine serum albumin (BSA, Gbcbio, China). Membranes were probed with anti-p-MLC (#3674T, 1:1000, CST, USA), anti-MLC (#3672S, 1:1000, CST, USA), anti-p-pkcα (#sc-377565, 1:500, SantaCruz, USA), anti-pkcα (#2056S, 1:1000, CST, USA), anti-MLCK (#BM4290, 1:400, BOSTER, China), anti-Calmodulin (#bs-3666R, 1:500, Bioss, China), and anti-actin (#66009-1-Ig, 1:6000, Proteintech, China), followed by horseradish-peroxi-dast-conjugated goat anti-rabbit IgG secondary antibody (#SA00001-2, 1:6000, Proteintech, China) or horseradish-peroxi-dast-conjugated goat anti-mouse IgG secondary antibody (#SA00001-1, 1:6000, Proteintech, China) for 1 h. The blots were detected using an ECL chemiluminescence substrates kit (#WBKLS0100, Millirore, USA).

### Statistical Analysis

All statistical analyses were performed using SPSS 20.0 (SPSS Inc., Chicago, IL, USA). The data are expressed as mean ± SD. Comparisons between groups were made by one-way analysis of variance (ANOVA) followed by Dunnett's test for separate comparisons. *P* < 0.05 was considered statistically significant.

## Data Availability Statement

The original contributions presented in the study are included in the article/Supplementary Material, further inquiries can be directed to the corresponding author/s.

## Author Contributions

YH and CW contributed to the design of the study. YH and JW carried out the majority of the experiments and statistical analyses. MF, JL, and XL contributed to the experimental work. BZ and WZ provided their expertise. All authors reviewed the draft and approved the submitted version.

## Conflict of Interest

The authors declare that the research was conducted in the absence of any commercial or financial relationships that could be construed as a potential conflict of interest.

## Supplementary Material

Supplementary Figure 1 Supplementary data for Figure 3. Three independent CO-IP experiments confirm the interaction between EspF protein and ANXA6 protein **(A)** and the C-terminal domain of EspF protein that interacts with ANXA6 protein **(B)**. M, protein marker.

Supplementary Figure 2 Supplementary data for Figure 4. Three independent western blot experiments to detect occludin and ZO-1 proteins in Caco-2 cells. E, cells transfected with pEGFP; EE, cells transfected with pEGFP-EspF; YA, cells transfected with pEYFP-Anxa6; CO, cells co-transfected with pEGFP-EspF and pEYFP-Anxa6; NC, non-transfected cells.

Supplementary Figure 3 Supplementary data for Figure 5. Three independent western blot experiments to detect occludin and ZO-1 protein in Caco-2 cells. NC, non-transfected cells; EY, cells transfected with pEYFP; EYEA, cells transfected with pEYFP-EspF-T2A-ANXA6; EE, cells transfected with pEGFP-EspF.

Supplementary Figure 4 Supplementary data for Figure 6. Three independent western blot experiments to detect MLCK-MLC signaling proteins in Caco-2 cells. NC, non-transfected cells; EY, cells transfected with pEYFP; EYEA, cells transfected with pEYFP-EspF-T2A-ANXA6; EE, cells transfected with pEGFP-EspF.

Supplementary Figure 5 Supplementary data for Figure 7. Three independent western blot experiments to detect occludin and ZO-1 proteins in Caco-2 cells. Cells were infected with EHEC EDL933w strains (WT), *espF*-deficient strains (Δ*espF*), *espF*-complementation Strains (p-Δ*espF*), DH5α strains (DH5α), respectively. UI, uninfected cells.

Supplementary Figure 6 The expression level of endogenous ANXA6 protein in Caco-2 cells. **(A)** The expression level of endogenous ANXA6 protein in non-transfected cells (N), cells transfected with pEYFP (EY), and cells transfected with pEYFP-EspF-T2A-Anxa6-HA (EYEA). M: protein marker. **(B)** Three independent western blot experiments to detect ANXA6 protein in non-transfected cells (N), cells transfected with pEGFP (E), cells transfected with pEGFP-EspF (EE), cells transfected with pEGFP-EspF/N (E-EN), cells transfected with pEGFP-EspF/C (E-EC). M: protein marker.

Supplementary Table 1 Formula of FRET method.
