# Supplementary figures and images for: Enterohemorrhagic Escherichia coli Effector Protein EspF Interacts With Host Protein ANXA6 and Triggers Myosin Light Chain Kinase (MLCK)-Dependent Tight Junction Dysregulation
Source: Front Cell Dev Biol. 2020 Dec 23;8:613061. doi: 10.3389/fcell.2020.613061 (PMC7785878; doi:10.3389/fcell.2020.613061)

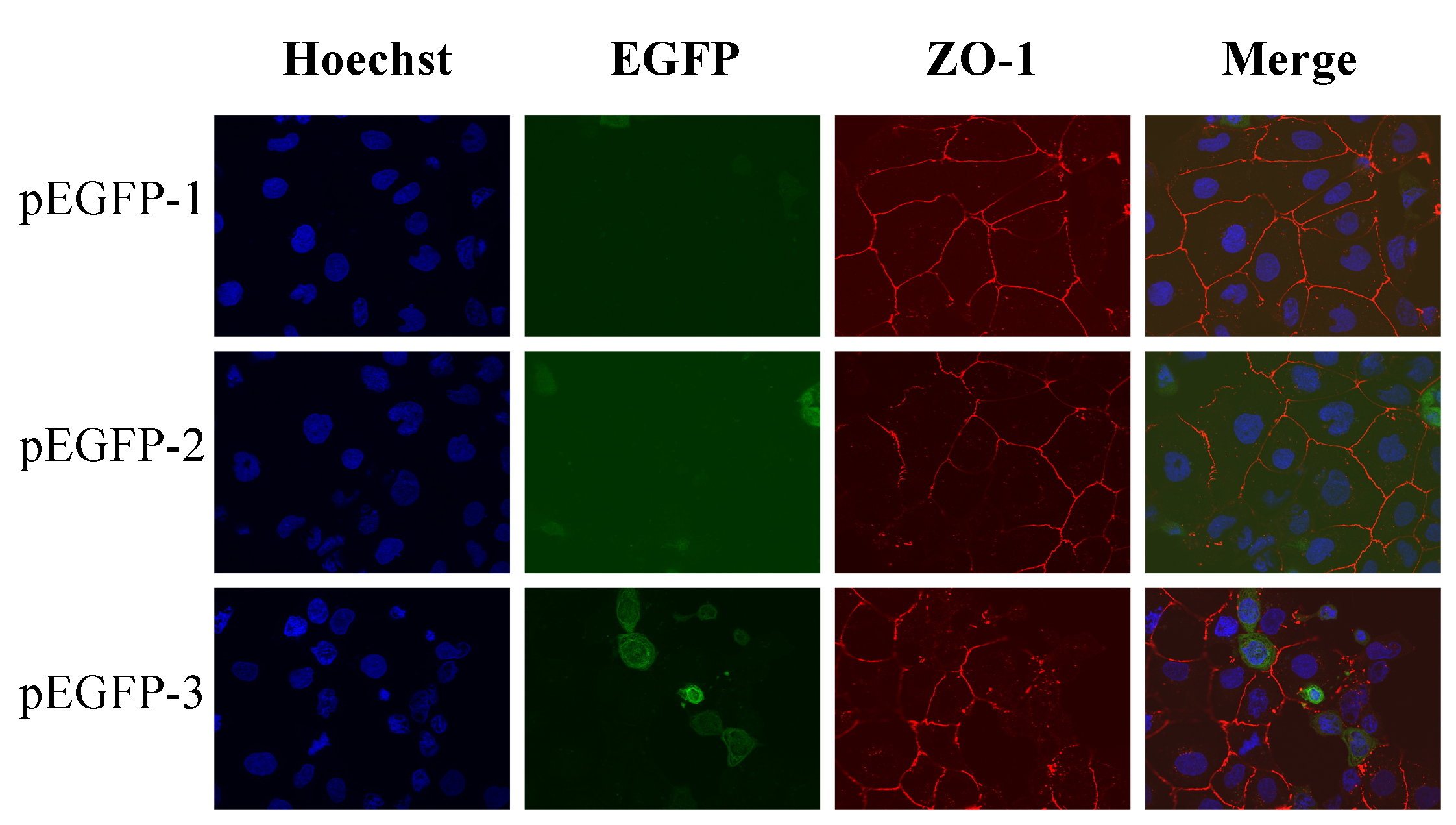

Supplement: Supplementary file 1 [file Image_7.TIF]

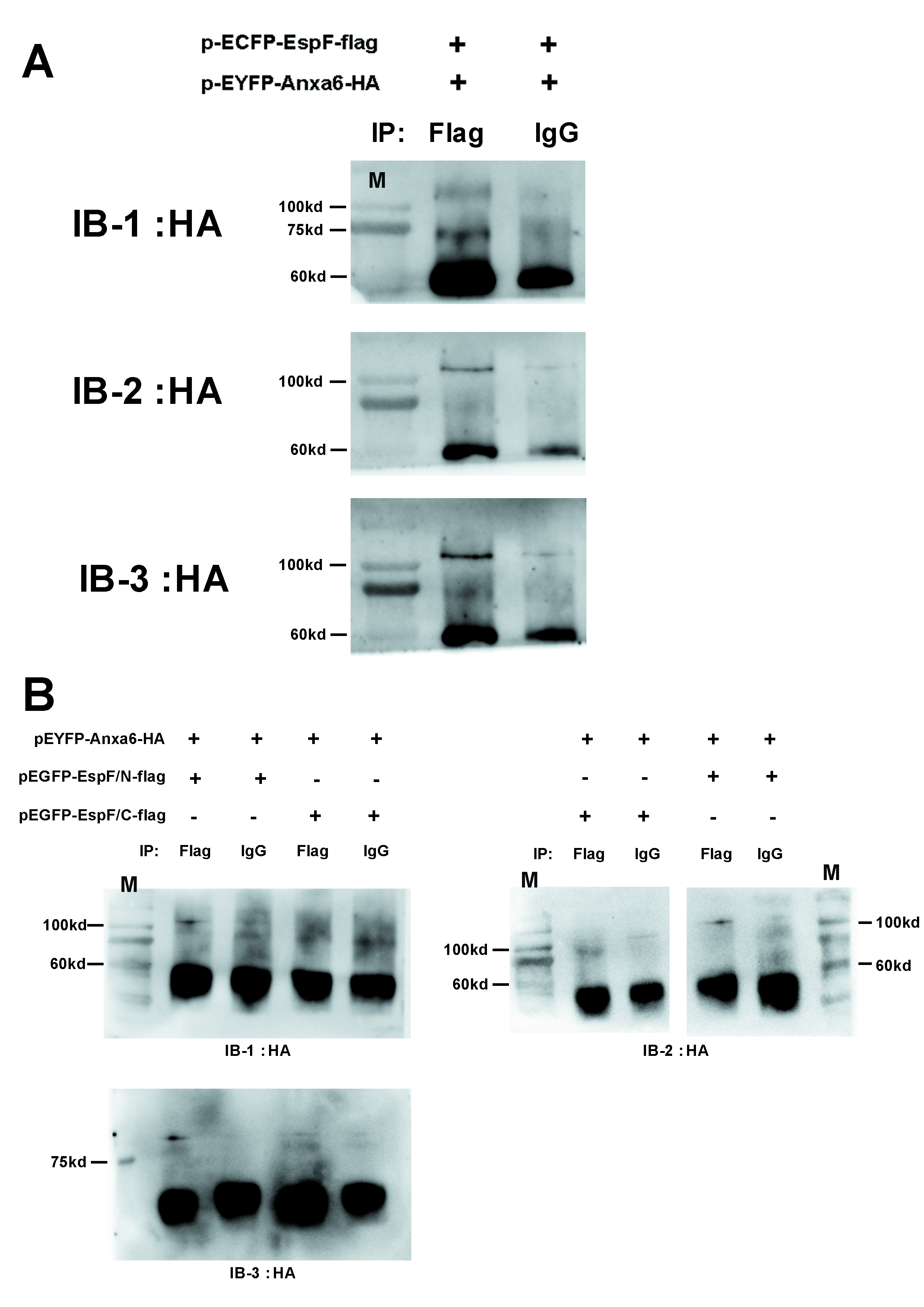

Supplement: Supplementary Figure 1 — Supplementary data for Figure 3. Three independent CO-IP experiments confirm the interaction between EspF protein and ANXA6 protein (A) and the C-terminal domain of EspF protein that interacts with ANXA6 protein (B). M, protein marker. [file Image_1.TIF]

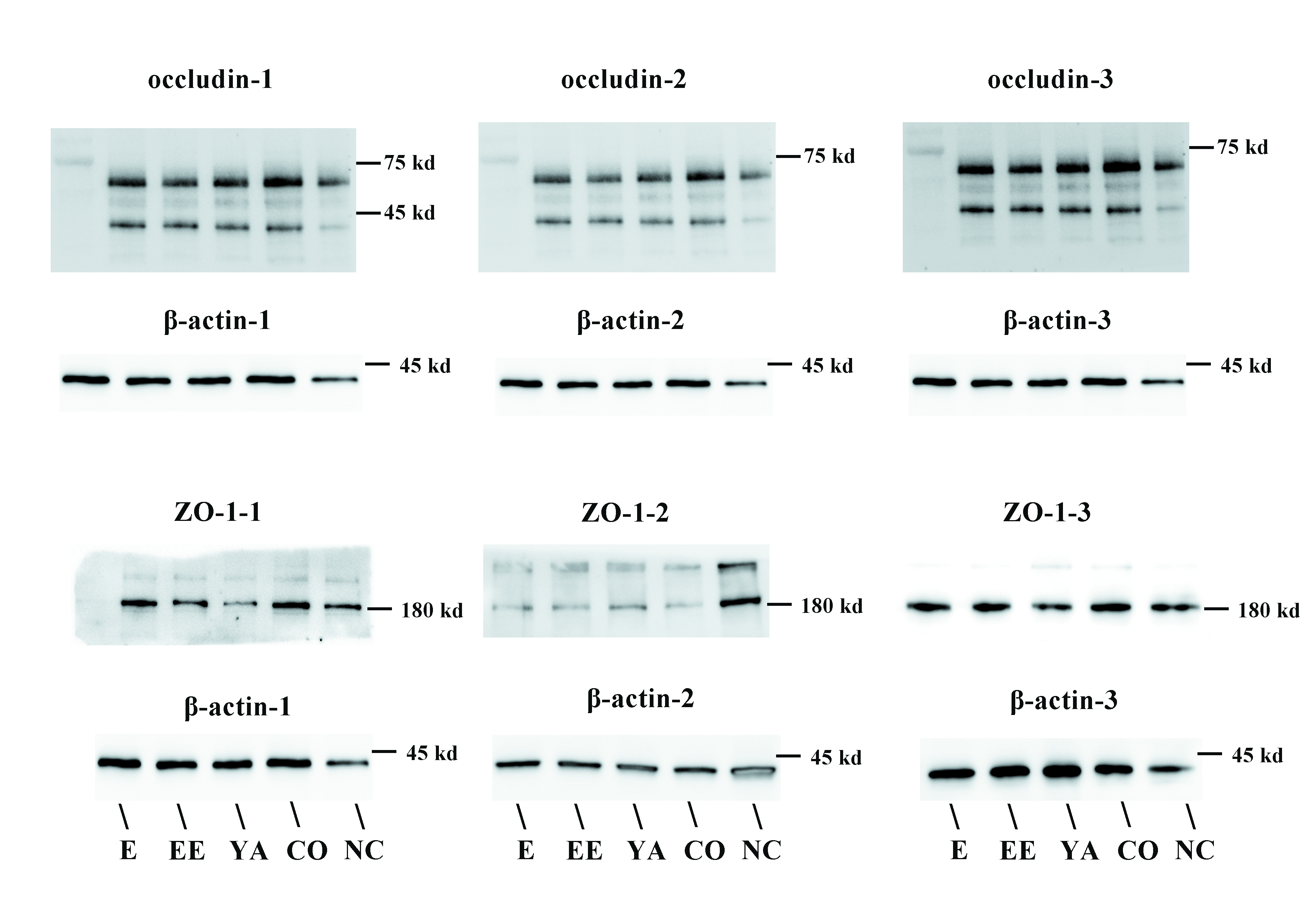

Supplement: Supplementary Figure 2 — Supplementary data for Figure 4. Three independent western blot experiments to detect occludin and ZO-1 proteins in Caco-2 cells. E, cells transfected with pEGFP; EE, cells transfected with pEGFP-EspF; YA, cells transfected with pEYFP-Anxa6; CO, cells co-transfected with pEGFP-EspF and pEYFP-Anxa6; NC, non-transfected cells. [file Image_2.TIF]

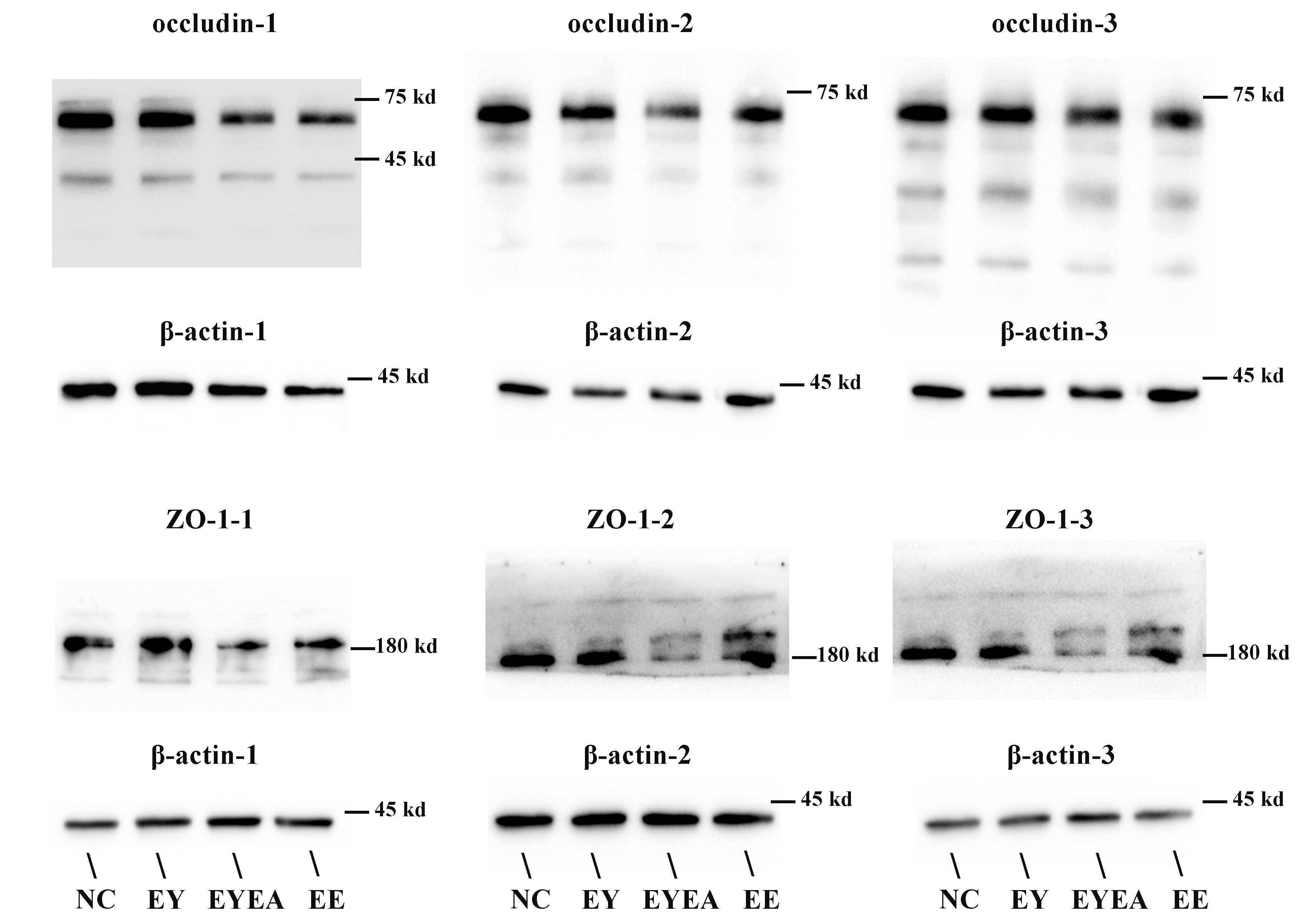

Supplement: Supplementary Figure 3 — Supplementary data for Figure 5. Three independent western blot experiments to detect occludin and ZO-1 protein in Caco-2 cells. NC, non-transfected cells; EY, cells transfected with pEYFP; EYEA, cells transfected with pEYFP-EspF-T2A-ANXA6; EE, cells transfected with pEGFP-EspF. [file Image_3.TIF]

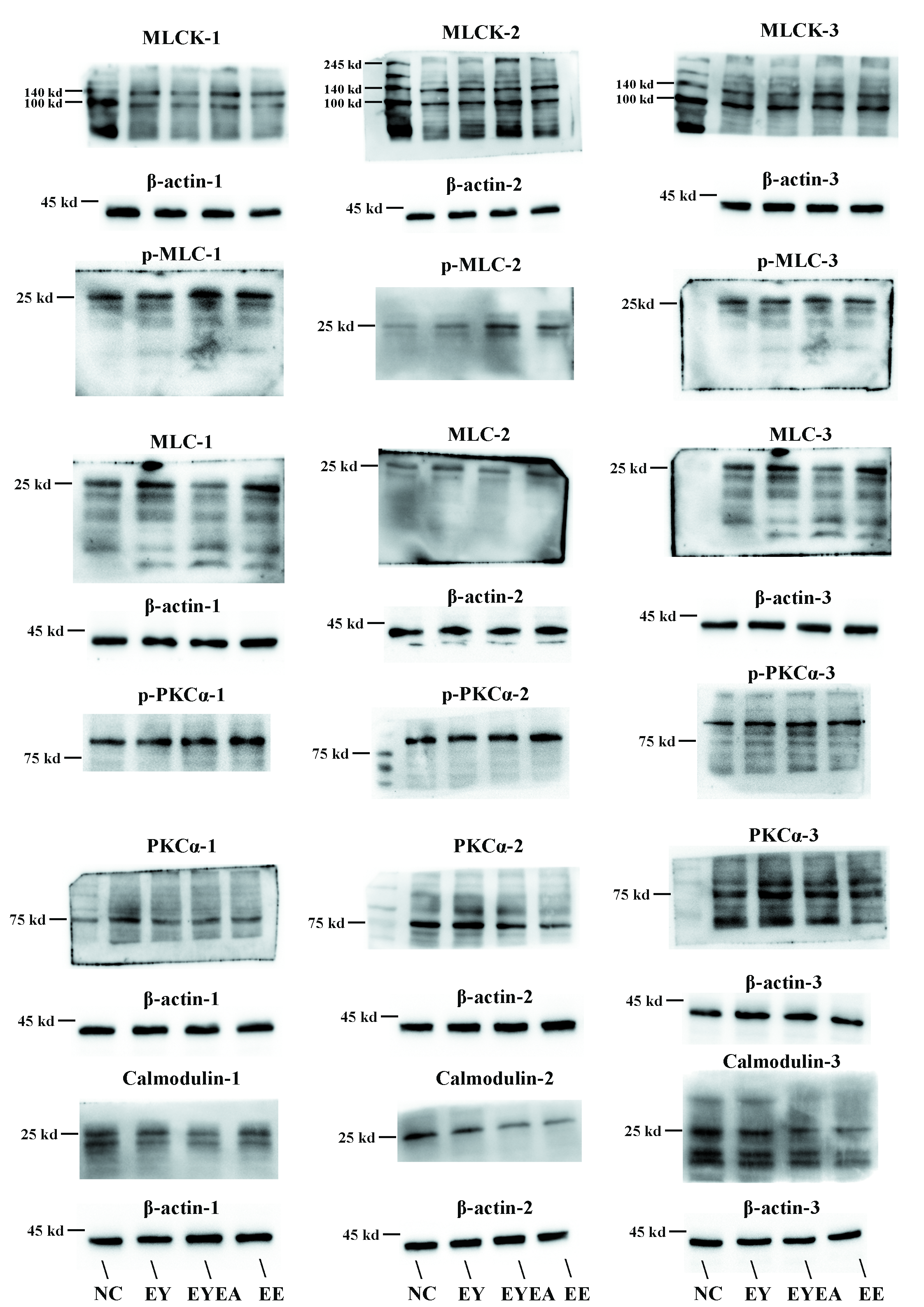

Supplement: Supplementary Figure 4 — Supplementary data for Figure 6. Three independent western blot experiments to detect MLCK-MLC signaling proteins in Caco-2 cells. NC, non-transfected cells; EY, cells transfected with pEYFP; EYEA, cells transfected with pEYFP-EspF-T2A-ANXA6; EE, cells transfected with pEGFP-EspF. [file Image_4.TIF]

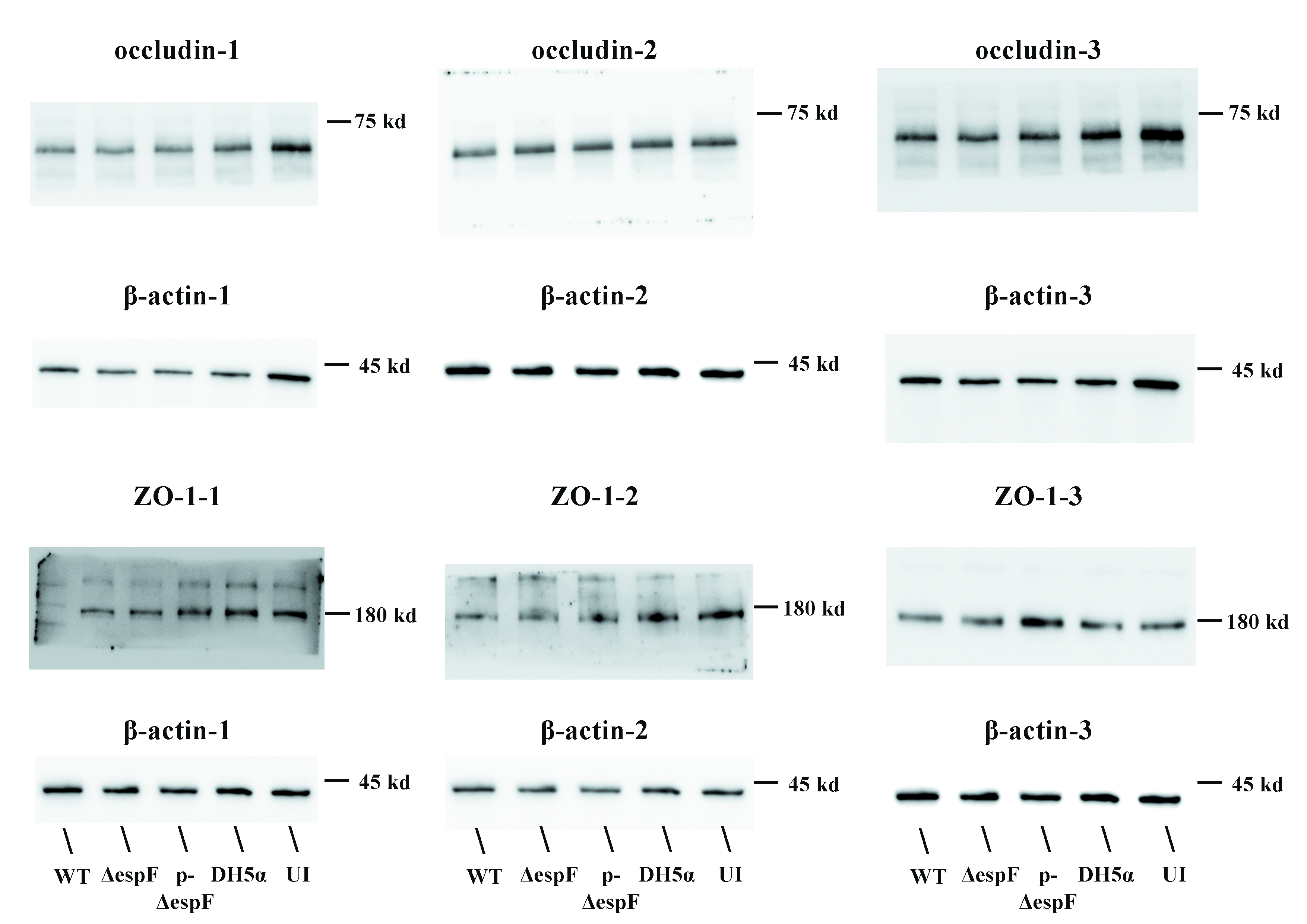

Supplement: Supplementary Figure 5 — Supplementary data for Figure 7. Three independent western blot experiments to detect occludin and ZO-1 proteins in Caco-2 cells. Cells were infected with EHEC EDL933w strains (WT), espF-deficient strains (ΔespF), espF-complementation Strains (p-ΔespF), DH5α strains (DH5α), respectively. UI, uninfected cells. [file Image_5.TIF]

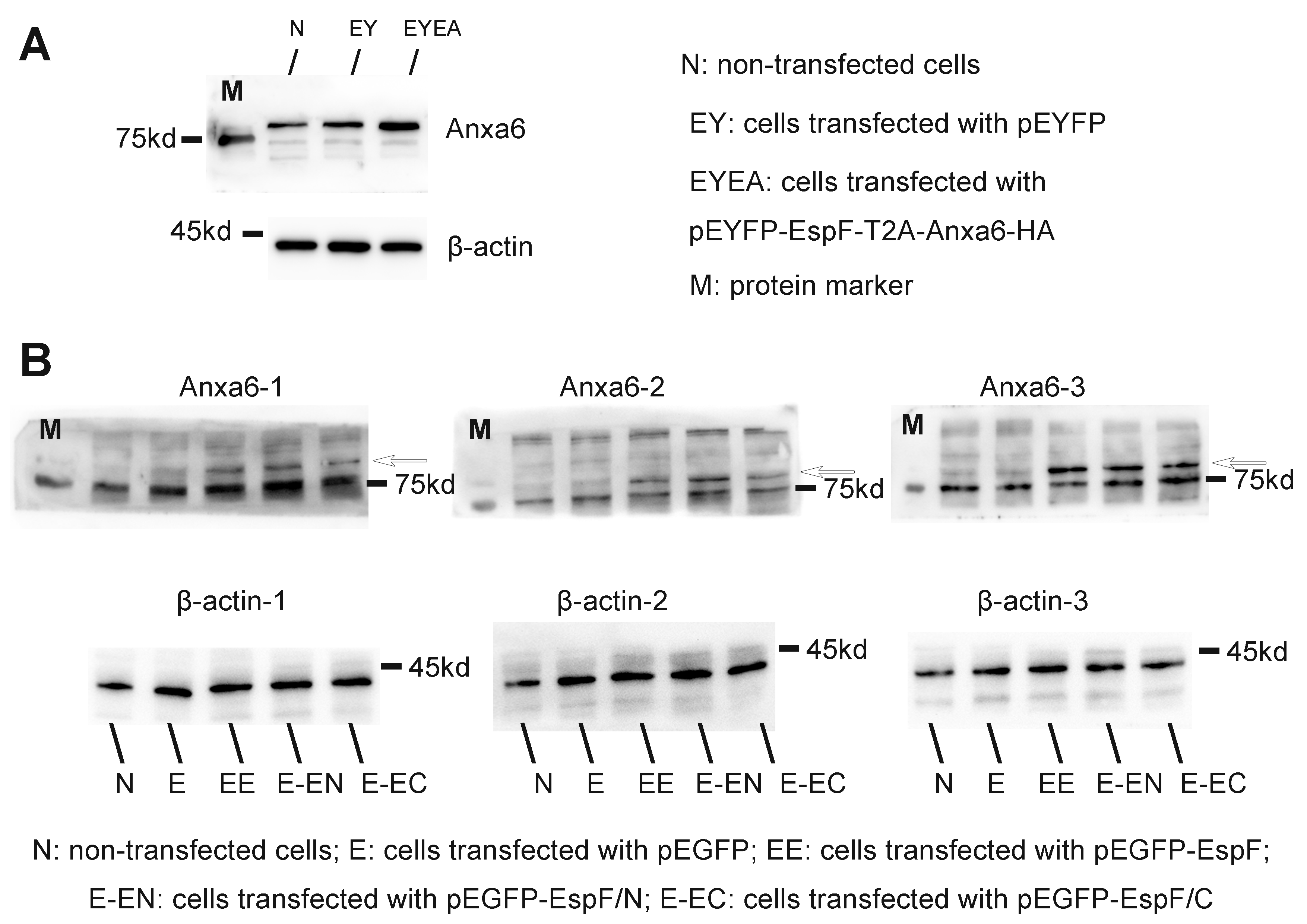

Supplement: Supplementary Figure 6 — The expression level of endogenous ANXA6 protein in Caco-2 cells. (A) The expression level of endogenous ANXA6 protein in non-transfected cells (N), cells transfected with pEYFP (EY), and cells transfected with pEYFP-EspF-T2A-Anxa6-HA (EYEA). M: protein marker. (B) Three independent western blot experiments to detect ANXA6 protein in non-transfected cells (N), cells transfected with pEGFP (E), cells transfected with pEGFP-EspF (EE), cells transfected with pEGFP-EspF/N (E-EN), cells transfected with pEGFP-EspF/C (E-EC). M: protein marker. [file Image_6.TIF]
